# The STAT6 inhibitor AS1517499 reduces the risk of asthma in mice with 2,4-dinitrochlorobenzene-induced atopic dermatitis by blocking the STAT6 signaling pathway

**DOI:** 10.1186/s13223-022-00652-8

**Published:** 2022-02-17

**Authors:** Xueying Li, Zhaoqing Han, Feng Wang, Jianou Qiao

**Affiliations:** 1 Department of Respiratory, Shanghai Ninth People’s Hospital Affiliated Shanghai JiaoTong University School of Medicine, No.639, Zhizaoju Road, Shanghai, 200001 China; 2 Department of Thoracic Surgery, Shanghai Ninth People’s Hospital Affiliated Shanghai JiaoTong University School of Medicine, No.639, Zhizaoju Road, Shanghai, 200001 China

**Keywords:** Atopic dermatitis, Asthma, STAT6, Th2 cells, Treg cells, AS1517499

## Abstract

**Background:**

Epidemiological studies have revealed a link between atopic dermatitis (AD) and asthma. AS1517499, a selective signal transducer and activation of transcription 6 (STAT6) inhibitor, has been shown to effectively block this connection. In this study, we further explored the underlying mechanism by constructing an AD mouse model.

**Methods:**

Female BALB/c mice were randomly divided into four groups (n = 10/group). The AD mouse model was established by 2,4-dinitrochlorobenzene induction with repeated ovalbumin challenge. AS1517499 and corn oil were used as treatment interventions. The features of airway inflammation, remodeling, and hyperactivity were analyzed.

**Results:**

Active use of AS1517499 in AD mice effectively reduced Th2-related cytokine levels, alleviated airway eosinophil and lymphocyte infiltration, and regulated GATA3/Foxp3 levels and subepithelial collagen deposition. These changes might be due to specific blockade of the STAT6 signaling pathway.

**Conclusion:**

AS1517499 could partially block the association between AD and asthma by specifically inhibiting the STAT6 signaling pathway.

## Background

Asthma is a complex chronic airway inflammatory disease characterized by airway inflammation and hyperresponsiveness. It is the result of genetic susceptibility and environmental factors and has obvious seasonal influence [1]. The prevalence of childhood asthma increased annually between 1999 and 2001 and gradually reached a plateau in 2013 [2]. According to the National Review of Asthma Deaths, 28 children died of asthma between February 2012 and January 2013 in the UK alone [3].

Atopic dermatitis (AD) is a chronic inflammatory skin disease, once known as atopic eczema, and is the most common form of chronic skin inflammation in children [4]. The worldwide prevalence of AD is between 1 and 20%, and the majority of cases reported onset before the age of 2 years [5]. Epidemiological studies have shown that the prevalence of allergic asthma and allergic rhinitis are increased in children if they suffer from AD before 5 years of age [6]. This disease progression from AD in infants to allergic asthma or asthma in children is termed the “allergic march.” The early onset and severity of AD are closely related to the development of an allergic march [7, 8]. In recent years, researchers have made increasing efforts for clarifying the possible pathogenesis of the allergic march, from nutritional factors and environmental factors to genes [9, 10], but to no avail.

AS1517499, a novel low molecular weight compound, is a derivative of 2-{[2-(4-hydroxyphenyl)ethyl]amino}pyrimidine-5-carboxamide and has been reported to be the most potent inhibitor of signal transducer and activator of transcription 6 (STAT6) [11]. AS1517499 might be useful for the treatment of various allergic conditions caused by an excess Th2 response, such as asthma and atopic diseases [11]. Additionally, studies have confirmed the effectiveness of STAT6 inhibition in asthmatic mice [12]. A study by Chiba et al. [13] showed that AS1517499 could ameliorate antigen-induced bronchial hypercontractility in mice by inhibiting the upregulation of RhoA and IL-13 production induced by antigens, thus having potential use for asthma treatment. Another study indicated that the novel STAT6 inhibitor AS1517499 could reduce the preventive effects of apoptotic cell instillation on bleomycin-induced lung fibrosis by suppressing PPARγ expression [14]. All of these reports showed that this novel STAT6 inhibitor can enter cells and suppress STAT6 signaling. STAT6 is a member of the signal transduction activator family. The cytokines interleukin (IL)-4 and IL-13 activate STAT6 phosphorylation and promote Th2 cell differentiation [15]. In addition, STAT6 can regulate the expression of GATA-binding protein 3 (GATA3), and GATA3, in turn, can reactivate the development of a Th2 response in STAT6-deficient T cells, making it a key factor in the differentiation and function of Th2 cells [16, 17]. Research on STAT6-deficient mice found that the Th2 immune response, and consequently airway eosinophilia and airway hyperresponsiveness (AHR), do not develop with allergen stimulation [18]. Furthermore, a study on mice with 2,4-dinitrochlorobenzene (DNCB)-induced AD has also found a remarkable increase in STAT6 protein levels [13]. These results suggest that AS1517499 might reduce the risk of asthma in mice with AD by blocking the STAT6 signaling pathway.

In previous experiments, we successfully established a mouse model to verify the associations between AD and asthma [19]. In the present study, DNCB and ovalbumin (OVA) were used to construct a mouse model of AD. Moreover, the possible mechanisms by which AS1517499 reduces the risk of asthma were explored.

## Methods

### Animals

Specific pathogen-free female BALB/c mice (4–6-week-old, 16–20 g) were provided by the Shanghai Slake Laboratory Animal Company (Shanghai, China). The mice were fed standard laboratory rat food and drinking water in a timely manner and were housed at room temperature (23 °C) with relative humidity of 40%, with a 12:12 h light–dark cycle. The experimental scheme was approved by the Animal Ethics Committee of Shanghai Ninth People’s Hospital (No. HKDL [2018]504).

### Grouping

**Fig. 1:**
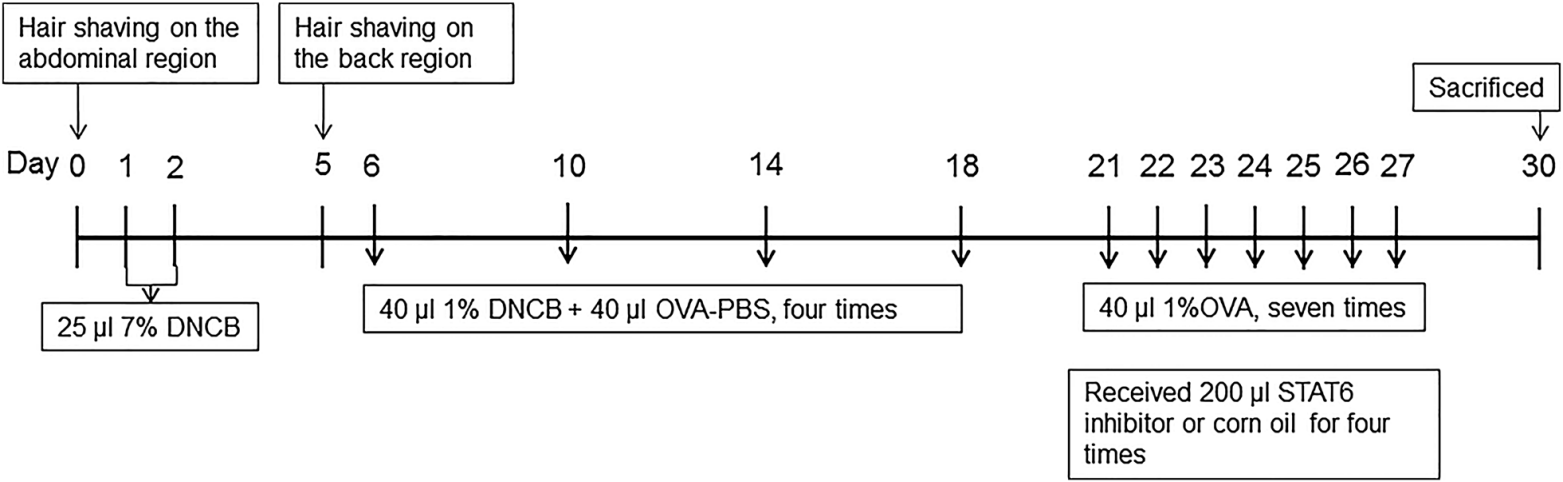
Experimental timeline of mouse model establishment and intervention. Forty female BALB/c mice were randomly divided into four groups (n = 10/group). The abdomens and backs of the mice were exposed to 20 μL of 7% 2,4-dinitrochlorobenzene (DNCB) for 2 consecutive days, and the back skin was additionally challenged with 40 μL of 1% DNCB and 40 μL of ovalbumin (OVA)-PBS solution every 3 days, for a total of four rounds. The control group was treated in the same manner with acetone solution. On day 21, the mice were challenged via inhalation of aerosolized 1% OVA solution for 30 min every day for 7 consecutive days. Intraperitoneal injection of AS1517499 (10 mg/kg; dissolved in corn oil) and corn oil were administered to the AD and control groups, respectively, 1 h before aerosol inhalation (days 21, 23, 25, and 27). On day 30, the mice were sacrificed, and the required samples were collected

Forty female BALB/c mice were fed adaptively for 1 week and were then randomly divided into four groups (n = 10/group) as follows: vehicle control (VC), AD, corn oil treatment (COTR), and AS1517499 treatment (AS) groups. The mice in all groups except the VC group received the identical DNCB and OVA treatment; specifically, DNCB (Shenzhen Chemical Co., Ltd., Guangdong, China) and OVA (Sigma V grade) were applied onto the back and abdomen to induce AD-like immunity and skin damage as described previously [19]. Briefly, a 2 × 2-cm area of abdominal hair was removed, and 25 μL of 7% DNCB solution was applied on days 1 and 2. From day 6 to 18, 40 μg of 1% DNCB solution and 40 μg of phosphate-buffered saline containing OVA (OVA-PBS solution, 40 μg OVA) were applied onto the exposed back skin every 4 days (four times; days 6, 10, 14, and 18). The VC group was treated similarly with acetone and OVA. Seventy-two hours after sensitization, the mice were placed in a transparent container of 15 × 20 × 20 cm and were challenged with 1% OVA aerosol daily for 30 min from day 21 to 27. At the same time, the mice in the AS and COTR groups were intraperitoneally injected with AS1517499 (100 nM; MCE, USA) and corn oil (200 μL), respectively, 1 h before aerosol inhalation (dose: 10 mg/kg) on days 21, 23, 25, and 27. The mice in the AD and VC groups were injected with acetone. AHR was assessed 72 h after the last aerosol inhalation. Tissues and cells were collected for further experimental analyses. The scheme for model construction is displayed in Fig. 1.

### Assessment of dermatitis severity

Severity scores were given for the following items, with scores comprising 0 (none), 1 (light), 2 (medium), and 3 (heavy): hemorrhagic erythema, scars, scratches, erosion, dryness, exfoliation, and swelling. The severity of dermatitis of the back and abdomen was scored twice per week. The total dermatitis severity score of mice in each group was calculated by summarizing the scores of each item [20].

### Airway compliance test

Whole body plethysmography was used to evaluate AHR in terms of changes in airway function after acetylcholine (ACH; Sigma) stimulation [21]. The plethysmograph consisted of a main chamber, which contained the conscious, spontaneous breathing animal, and a reference chamber. The pressure differences between the two chambers (box pressure signals) were measured. After baseline measurements with aerosolized saline, the mice were challenged with ACH (4, 8, 16, 32, and 64 mg/mL) for 3 min, and the average readings from each group were recorded. The dimensionless parameter enhanced pause (Penh) was used to represent the data.

### Sample preparation

The mice were anesthetized via intraperitoneal injection of 1% pentobarbital sodium. Blood samples were collected by eyeball extraction and centrifuged for 10 min at 3000 rpm. The upper serum layer was collected and stored at − 80 °C. With the mice supine, the bronchoalveolar lavage fluid (BALF) was collected and was then centrifuged at 4 °C for 10 min, at 2000 rpm. The supernatant was discarded and the pellet was precipitated with white blood cell diluent. The skin lesions and the right upper lung were dissected and fixed in formalin with 4% paraformaldehyde for 24 h. They were then paraffin-embedded for histological analyses. The remaining right lung tissues were placed in a cryopreservation tube and stored in liquid nitrogen for future use.

### BALF cell collection and counting

The BALF precipitate was resuspended in 0.5 mL of PBS. To perform the cell counts, a 10-μL cell sample was transferred to a hemocytometer, and the number of cells in four squares was counted. The total number of cells was calculated according to the following formula: cell number/mL = [(number of cells in four squares/4) × 10^4^]. The smears were dried and stained with Wright-Giemsa stain (Wuhan Servicebio Co., Ltd., Hubei, China). The eosinophils (Eos), lymphocytes (Lyms), macrophages, and neutrophils (Neus) recovered from 300 cases were counted blindly according to morphological criteria [22]. The final results are expressed in absolute cell numbers.

### Histopathological measurements

After the paraffin-embedded skin tissues were sectioned and stained with hemoxylin-eosin (H&E), the inflammatory changes were observed at 20 × magnification. Similarly, the right lung tissue was stained with H&E and Masson trichrome (Wuhan Servicebio Biological Technology Co., Ltd, Hubei, China) to observe the features of lung tissue structure, inflammatory cell infiltration, and collagen proliferation.

### Serum cytokine measurement

The serum aliquots were stored at − 80 °C until cytokine analysis, during which the concentrations of IL-10 and IL-33 were measured using the corresponding ELISA Kit (Elabscience, Houston, TX, USA).

### Detection of mRNA levels of *STAT6*, *GATA3*, and *Foxp3* using RT-PCR

TRIzol (Takara, Japan) was used to extract RNA from tissues. The concentration and purity of total RNA was determined, and the RNA was reverse-transcribed to cDNA. Primers (Shanghai Boshan Biotechnology Co., Ltd, Shanghai, China) were chosen based on the gene sequences published by GenBank and other related literature. The primers were amplified with the SYBR Green II fluorescence kit (Takara, Japan), and an ABI7500 real-time PCR system (Thermo Fisher) was used for this experiment. *GAPDH* was used as the internal reference gene. The cycle threshold (CT) value (inflection point of amplified power curve) was obtained. The relative expression of target genes was then calculated using the 2^−ΔΔCT^ method [23].

### Detection of protein levels of STAT6, GATA3, and Foxp3 using western blotting

Protein was extracted from lung tissues and its concentration was determined using a bicinchoninic acid kit. The tissue was added into sample buffer and boiled for 10 min. Protein separation was then achieved by 10% polyacrylamide gel electrophoresis, conducted at 80 V for 30 min and at 120 V thereafter. The protein was then transferred to a polyvinylidene fluoride membrane with a current of 250 mA for 2 h and incubated at room temperature with 5% milk for 2 h and with a primary antibody (1:2000) overnight at 4 °C. The blots were washed thrice (10 min each) with tris-buffered saline Tween-20 (TBST) and incubated with a secondary antibody (1:10,000). Following incubation at room temperature for 1 h, the blot was rinsed three times (10 min each) with TBST and developed using chemiluminescence reagents. GAPDH was used as the internal reference. The target protein signals were analyzed using Image J.

### Data analysis

All data are expressed as the mean ± standard deviation (SD) and were analyzed using the SPSS23.0 software. Comparisons among groups were assessed using one-way ANOVA and the Student–Newman–Keuls test. Statistical significance was considered when *P* < 0.05.

## Results

### AD-like skin lesions in BALB/c mice

The dermatitis severity scores of the AD and control groups before and after treatment are listed in Table 1. After DNCB sensitization, the total score in the AD group (16.2 ± 1.38) was significantly higher than that in the control group (0, *P* < 0.05). Hemorrhagic erythema, scars, dryness, edema, exfoliation, and erosion of the skin were observed in the AD group. Furthermore, the severity scores were significantly lower after treatment with AS1517499 (9.2 ± 1.73) compared to those with corn oil (15.8 ± 1.5).

**Table 1:** Evaluation of the AD-like dermatitis scoring system

Group	Number of mice	Score
VC	10	0
AD	10	16.2 ± 1.38*
COTR	10	15.8 ± 1.5*
AS	10	9.2 ± 1.73**

The total score involved the sum of seven clinical items: hemorrhagic erythema, scars, scratches, erosion, dryness, exfoliation, and swelling

The degree of each symptom was scored 0 (none), 1 (mild), 2 (moderate) or 3 (severe)

Scoring was done twice a week

The results are expressed as mean ± SD (n = 5)

*P < 0.05 indicates significant difference between the AD group and vehicle control (VC) groups

**P < 0.05 indicates significant difference between the AS and AD groups

### AD-like histopathological changes

**Fig. 2:**
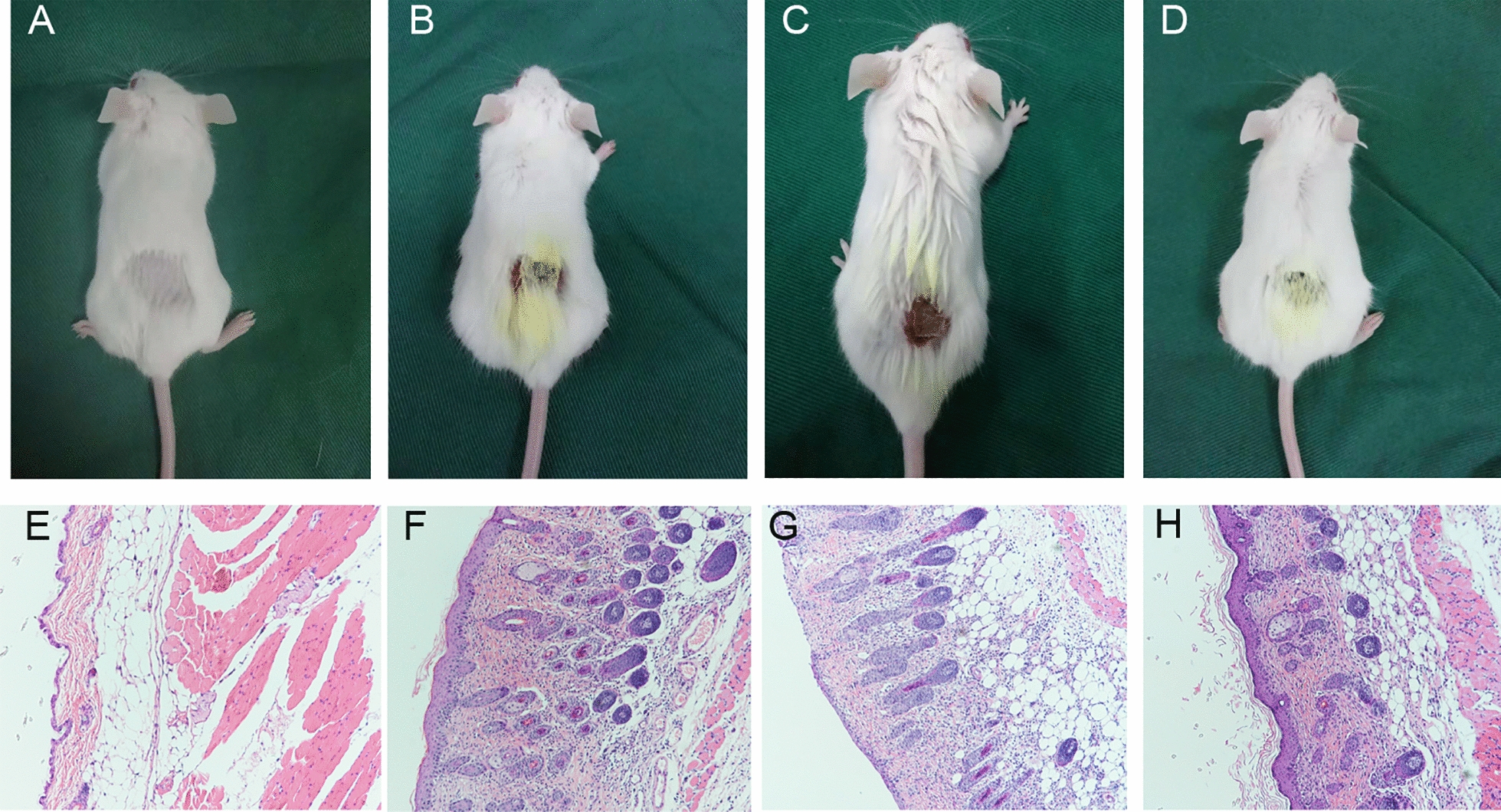
Effects of AS1517499 on the severity of skin and histopathological characteristics of 2,4-dinitrochlorobenzene (DNCB)-induced atopic dermatitis (AD) in BALB/c mice. Representative images acquired after treatment (upper panel), and AD-like histopathological changes revealed by H&E staining under a light microscope (20 × magnification) in mouse skin samples (below panel). The images are representatives of the following four groups (n = 10 per group): **A**\**E** vehicle control (VC), **B**\**F** AD, **C**\**G** corn oil treatment (COTR), and **D**\**H** AS1517499 treatment (AS) groups

Compared with the observations in the control group, the pathological sections of skin tissues in the AD mice showed marked hyperkeratosis and hypertrophy of the spinous layer, as well as increased infiltration of inflammatory cells, such as mast cells and Eos, in the dermis (Fig. 2). These skin lesions were significantly alleviated after treatment with AS1517499, and no significant changes were observed between the COTR and AD groups (Fig. 2).

### Detection of airway responsiveness in BALB/c mice

**Fig. 3:**
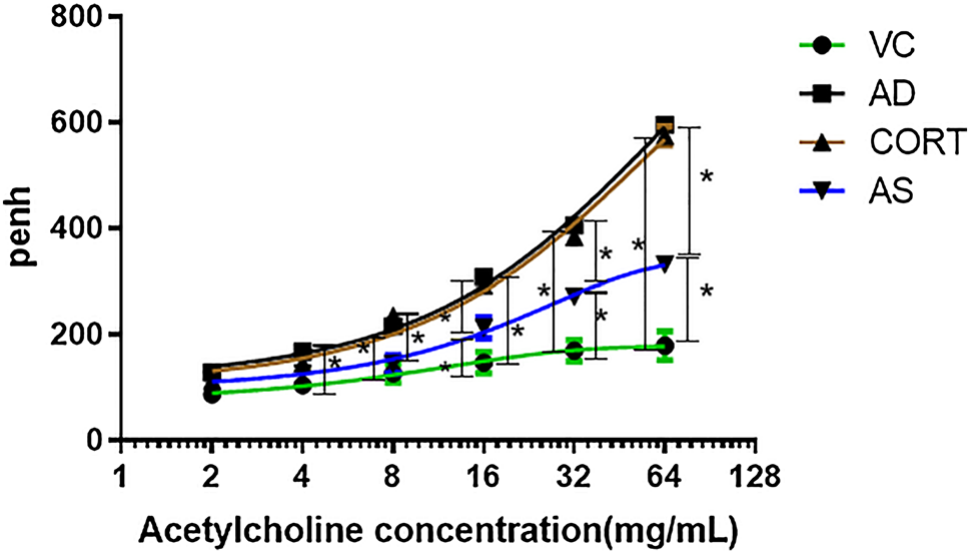
Measurement of airway hyperresponsiveness in acetylcholine (ACH)-challenged mice. The Penh values of the four groups were measured. The results are expressed as the mean ± SD. Vehicle control (VC), atopic dermatitis (AD), corn oil treatment (COTR), and AS1517499 treatment (AS) groups (n = 10 per group). *P < 0.05

During aerosol inhalation, mice in the AD and COTR groups had apparent frontal impatience, and nose scratches were more frequent relative to the numbers in the other two groups. After initiation of ACH aerosolization, the average Penh in the AD group showed a dose-dependent increase with increasing ACH exposure, and the overall increase was significantly higher than that in the control group (*P* = 0.026, Fig. 3). The extent of the increase from baseline Penh with AS1517499 treatment was lower than that in the AD group (*P* = 0.033), but the average increase was still significantly higher than that in the control group (*P* = 0.019, Fig. 3). There was no significant difference between the COTR and control groups (*P* = 0.416, Fig. 3).

### Number of inflammatory cells in BALF

**Fig. 4:**
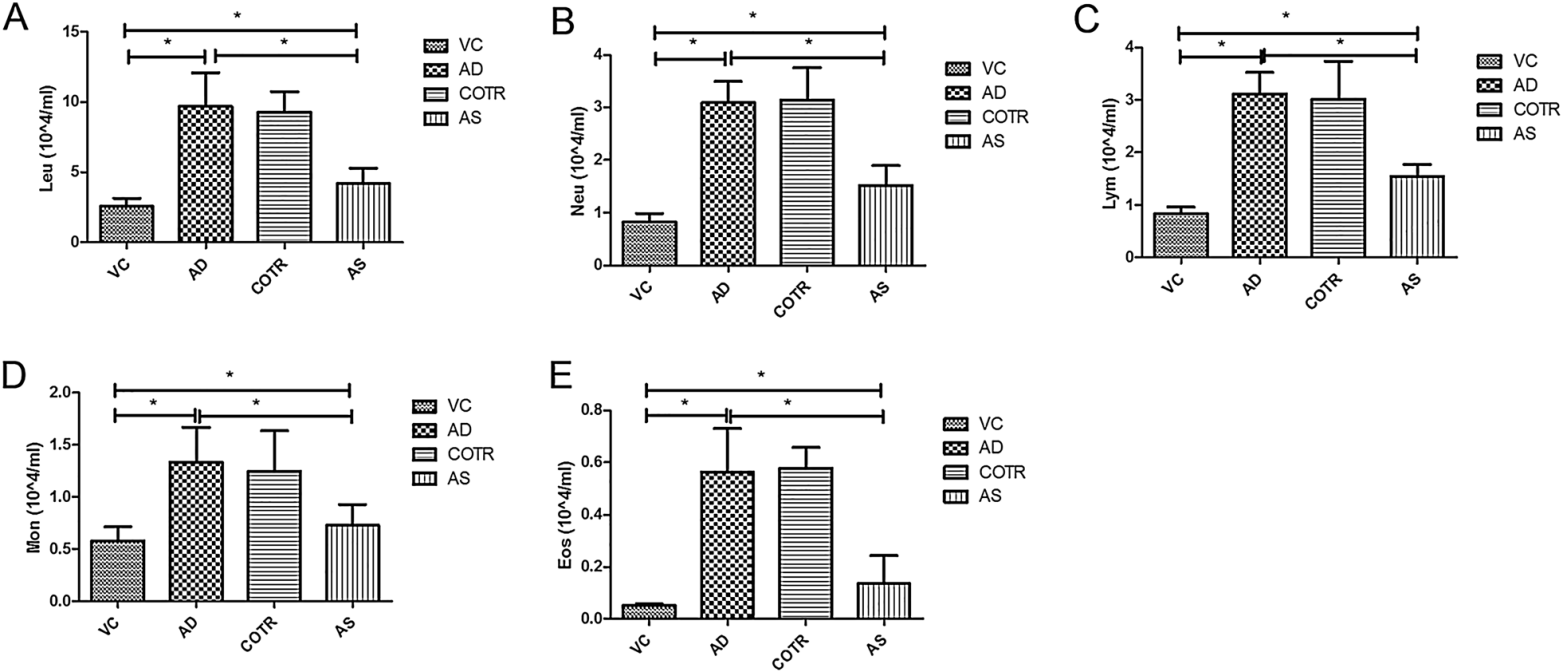
Cellular profile of bronchoalveolar lavage fluid. Leukocyte classification counting with Wright-Giemsa staining: **A** Total leukocyte count (Leu), **B** neutrophil (Neu), **C** lymphocyte (Lym), **D** monocyte (Mon), and **E** eosinophil (Eos) counts. The results are expressed as the mean ± SD (n = 10 per group). *P < 0.05

Compared with those in the control group, the total leucocyte numbers in the BALF of the AD group were significantly increased (*P* ≤ 0.0001); the number of Neus (*P* ≤ 0.0001), Lyms (*P* ≤ 0.0001), monocytes (*P* ≤ 0.0001), and Eos (*P* ≤ 0.0001) were also significantly increased (Fig. 4). These numbers decreased significantly after AS1517499 treatment (*P* ≤ 0.0001), but changes were not significant in the COTR group (Fig. 4).

### Histopathology of the lung

**Fig. 5:**
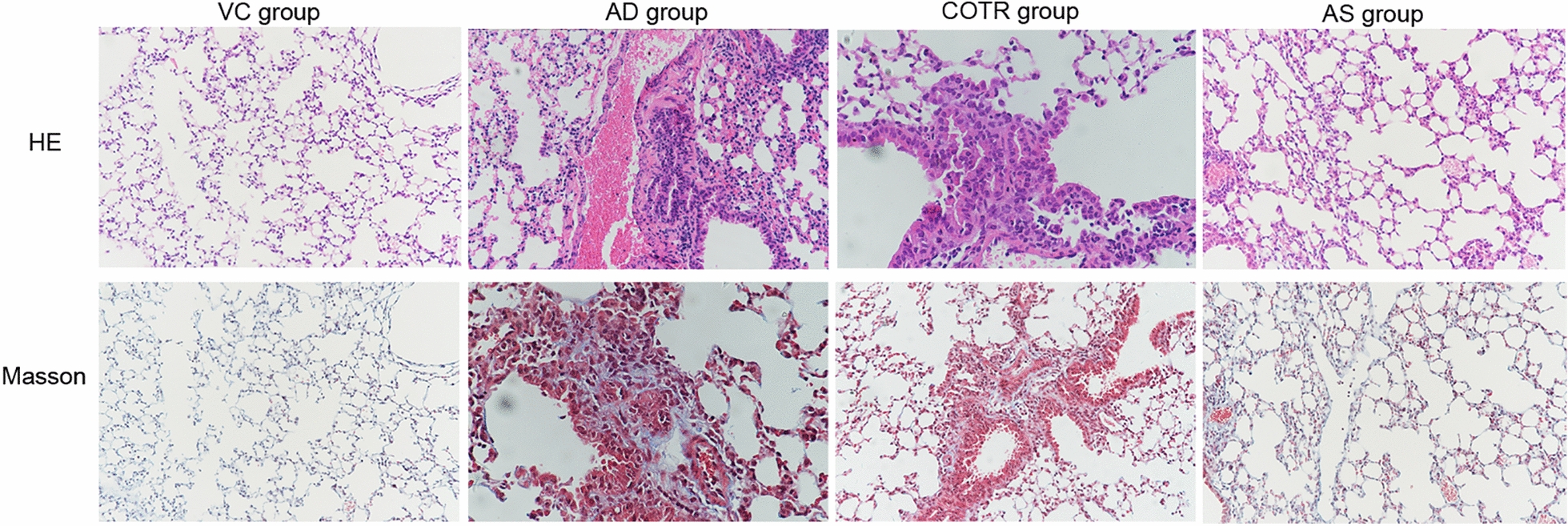
Histopathological analysis of airway structure and collagen distribution. Images of pathological sections of the lung tissues following H&E staining (upper panel) and Masson staining (lower panel) when observed under electron microscopy (100 × magnification). VC, vehicle control; COTR, corn oil treatment; AS, AS1517499 treatment (n = 10 per group)

H&E staining showed that compared to that in the control mice, infiltration of inflammatory cells around the alveoli and alveolar septum in AD mice increased; meanwhile, that around the bronchioles and alveolar septum in AS mice decreased compared with the levels in AD mice (Fig. 5). Additionally, Masson staining showed that the collagen content in the AD group was significantly higher than that in the VC group (Fig. 5). Collagen agglutination bundles and visible protein content in the AS group were significantly decreased relative to those in the AD group, whereas no significant changes in these pathological features were found between the COTR and AD groups (Fig. 5).

### ELISA of serum IL-10, IL-13, and IL-33 levels

**Fig. 6:**
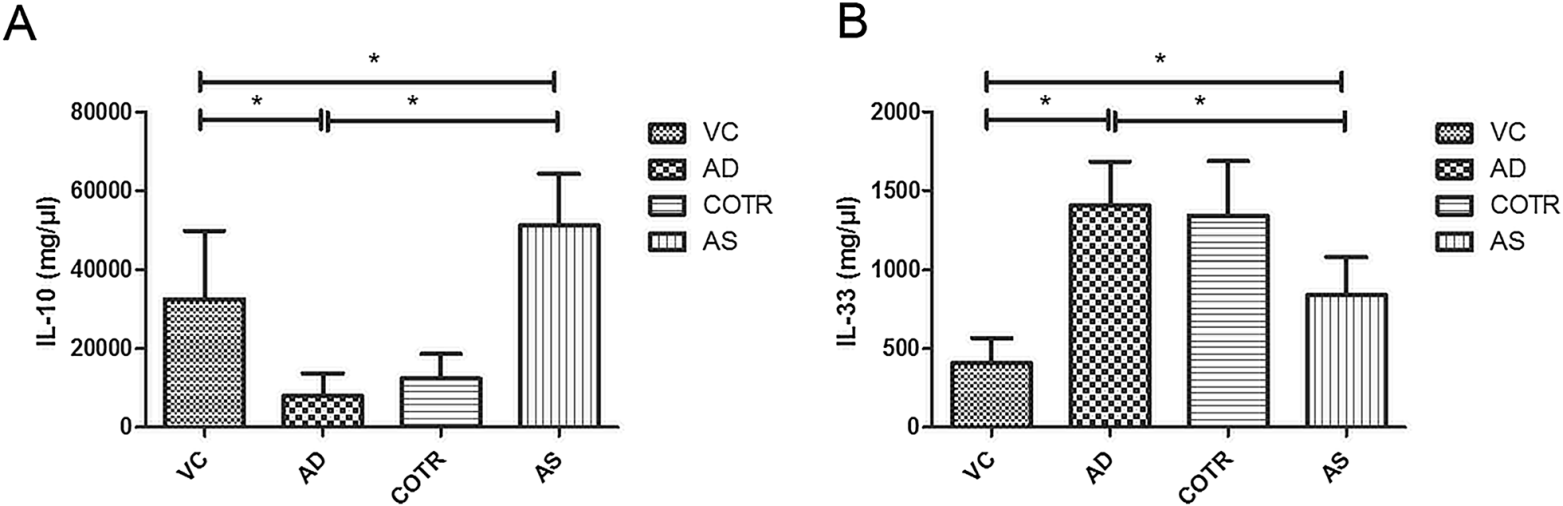
Serum levels of IL-10 and IL-33. ELISA results of **A** IL-10 and **B** IL-33 levels in the serum. The results are expressed as the mean ± SD (n = 10 per group). *P < 0.05

There were no significant differences in IL-10 and IL-33 levels between the AD and COTR groups (Fig. 6). Compared with levels in the VC group, serum IL-10 level was significantly reduced in the AD group (*P* < 0.05), and after AS1517499 treatment, its level was evidently increased (*P* ≤ 0.0001, Fig. 6A). Serum IL-33 level was significantly higher in the AD mice than in the control mice (*P* ≤ 0.0001); meanwhile, AS1517499 administration markedly ameliorated this increase caused by DNCB (*P* ≤ 0.0001, Fig. 6B). All of the results indicated that the repeated administration of DNCB increased the IL-33 level but inhibited IL-10 production, whereas the STAT6 inhibitor (AS1517499) significantly restored these levels.

### mRNA and protein levels of STAT6, GATA3, and Foxp3

**Fig. 7:**
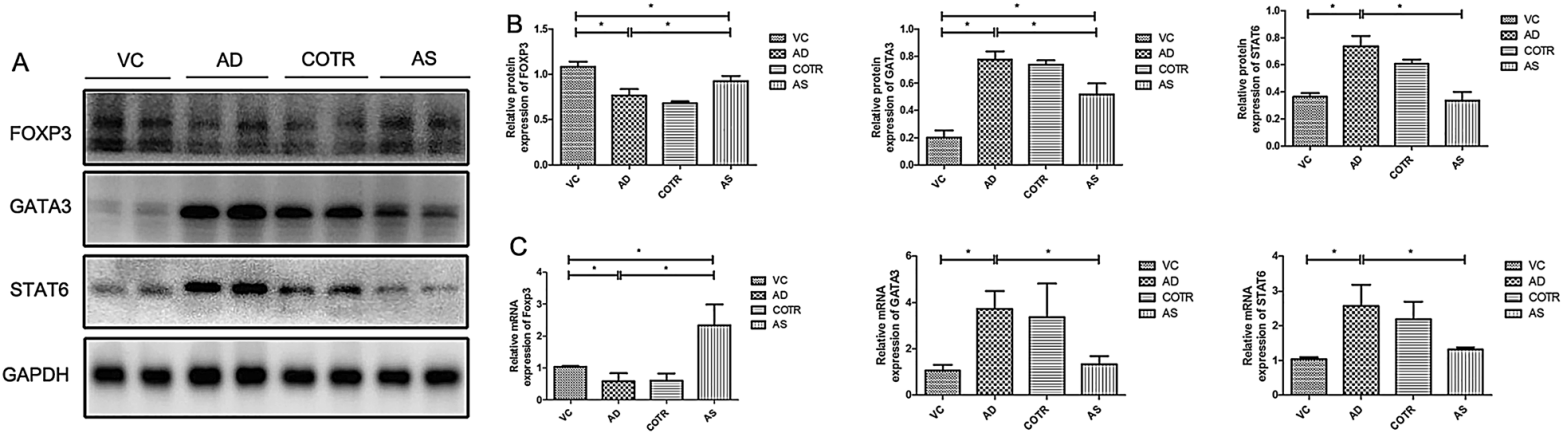
Expression of GATA3, Foxp3, and STAT-6 in lung tissues. The right lung tissue was obtained from the four groups of mice and analyzed using RT-qPCR and western blotting. **A** Protein expression analysis of Foxp3, GATA3, and STAT6 in mouse lung tissues; **B** Image J quantitative analysis of the western blot results. **C** Quantitative mRNA expression of *Foxp3*, *GATA3*, and *STAT6*. The levels of FOXP3, GATA3, and STAT6 were measured relative to those of GAPDH. All data are expressed as the mean ± SD (n = 10 per group). *P < 0.05

The protein expression of STAT6 and GATA3 in the AD group was significantly higher, whereas that of Foxp3 was significantly lower, relative to levels in the VC group (*P* ≤ 0.0001, Fig. 7A, B). The trend in *Foxp3*, *GATA3*, and *STAT6* mRNA expression in different groups, determined using RT-qPCR, was similar to that detected for the corresponding protein expression using western blotting (Fig. 7C).

## Discussion

Epidemiological investigations have shown that approximately 30–60% of children with AD eventually develop asthma or seasonal allergy disease [20]. However, the specific mechanism is not yet clear. Previously, we successfully established an AD mouse model for the study of asthma and detected elevated levels of IL-4, IL-5, and IgE, confirming the involvement of Th2 inflammation in the progression from AD to asthma [19]. Previous murine studies have shown that the main pathophysiological characteristics of asthmatic lungs are airway inflammation, AHR, and airway remodeling [24, 25]. Furthermore, Eos in the BALF are significantly increased in asthmatic mouse models [26]. Our results showed that BALF Neus, Lyms, Eos, and other inflammatory cells were significantly increased in the AD group. The lung tissue sections of AD mice also showed significant changes in lung tissue structure, with significantly higher collagen content than that in VC mice. After treatment with the STAT6 protein antagonist AS1517499, the skin lesions of mice were significantly alleviated and inflammatory cell infiltration and collagen content in lung tissues also significantly decreased. Therefore, it is reasonable to speculate that STAT6-related signaling pathways might be involved in the pathogenesis of AD and asthma.

IL-33 is a member of the IL family, localized in the nucleus of producing cells and secreted after cellular destruction [27]. It plays a biological role by binding to ST2 receptors [28]. IL-33 levels in the skin of patients with AD are increased significantly [29]. In keratinocytes, interferon (IFN)-R can stimulate the expression of IL-33 and its corresponding receptor ST2 [30]. IL-33 can further bind to ILC2S receptors and synergistically induce the production of IL-13 [31]. IL-13 is an important promoter of the STAT6 signaling pathway; it binds to IL-13R alpha 1 (IL-13Rα1) receptors, consequently leading to activated STAT6 proteins entering the nucleus and binding to target genes, inducing target gene expression [32]. A study by Yokozeki et al. showed that STAT6 proteins activated by IL-13 are crucial in the pathogenesis of AD [33]. Another study indicated that IL-33 from house dust mite extract-treated alveolar epithelial cells stimulates CD146 expression, promoting epithelial–mesenchymal transition during airway remodeling in chronic allergic inflammation [34]. Combined with our results, it can be inferred that AS1517499 could improve the symptoms of AD by regulating IL-33 expression, thus having an important effect on asthma.

A Treg/Th2 imbalance is one of the important mechanisms underlying asthma [35]. GATA3 and Foxp3 are specific transcription factors of Th2 and Treg cells, respectively [36]. GATA3 can bind directly to the promoter region of *Foxp3* and inhibit induction of the promoter gene, thereby reducing expression of the Treg cell-specific transcription factor Foxp3, in turn reducing the number of Treg cells and creating a Th2-dominated immune environment [37, 38]. STAT6 − / − mice are resistant to airway inflammation, with significantly increased Treg cells in vivo. The STAT6 signaling pathway also participates in the differentiation of Th2 cells mediated by IL-13 [39]. STAT6 also regulates the differentiation of Th2 cells and Treg cells by regulating the expression level of the Th2-specific transcription factor GATA3 [40]. Our data showed that GATA3 expression in the lungs of AD mice was increased significantly, whereas Foxp3 expression was decreased, alongside a decrease in Treg-related IL-10 levels. After the intraperitoneal injection of AS1517499, the expression of GATA3 decreased significantly, whereas Foxp3 and IL-10 expression increased. The severity of skin lesions was relieved, which was accompanied by significant alleviation of airway inflammation, hyperresponsiveness, and remodeling.

## Conclusions

The STAT6 signaling pathway might be involved in the pathogenesis of asthma development associated with AD. Our results provide insights into the underlying molecular mechanisms of asthma development in the context of AD. However, our findings have not been validated in humans and they have not been further analyzed at the cellular level.

## Data Availability

The data used to support the findings of this study are available from the corresponding author upon request.

## Abbreviations

AD: Atopic dermatitis
STAT6: Signal transducer and activator of transcription 6
OVA: Ovalbumin
GATA3: GATA-binding protein 3
DNCB: Dinitrochlorobenzene
VC: Vehicle control
COTR: Corn oil treatment
AHR: Airway hyperresponsiveness
ACH: Acetylcholine
BALF: Bronchoalveolar lavage fluid
Eos: Eosinophils
Lyms: Lymphocytes
Neus: Neutrophils
H&E: Hematoxylin–eosin
CT: Cycle threshold
IFN: Interferon
IL-13Rα1: IL-13R alpha 1
